# Quality of Malaria Treatment Provided under ‘Better Health Together’ Project in Ethnic Communities of Myanmar: How Are We Performing?

**DOI:** 10.3390/tropicalmed4040140

**Published:** 2019-12-04

**Authors:** Phyo Wai Minn, Hemant Deepak Shewade, Nang Thu Thu Kyaw, Khaing Hnin Phyo, Nay Yi Yi Linn, Myat Sandi Min, Yan Naing Aung, Zaw Toe Myint, Aung Thi

**Affiliations:** 1 Community Partners International, Yangon 11201, Myanmar; january.dr.sandi@gmail.com (M.S.M.); yannaing.aung128@gmail.com (Y.N.A.); zawtoemyint@cpintl.org (Z.T.M.); 2 International Union against Tuberculosis and Lung Disease (The Union), 75006 Paris, France; hemantjipmer@gmail.com; 3 The Union South East Asia Office, New Delhi 110016, India; 4 Karuna Trust, Bengaluru 560041, India; 5 The Union Myanmar Country Office, Mandalay 05021, Myanmar; nangthu82@gmail.com (N.T.T.K.); khainghninphyo1990@gmail.com (K.H.P.); 6 Vector Borne Disease Control Program, Department of Public Health, Ministry of Health and Sports, Nay Pyi Taw 15011, Myanmar; nayyiyilinn@gmail.com (N.Y.Y.L.); aungthi08@gmail.com (A.T.)

**Keywords:** ethnic minorities, hard-to-reach areas, early diagnosis and treatment, management of malaria, undifferentiated fever, SORT IT

## Abstract

Malaria accounted for 18% of all deaths in the ethnic communities of Myanmar. In this cross-sectional study, we assessed the extent of and factors associated with receipt of quality malaria treatment services provided by integrated community malaria volunteer (ICMV) under six ethnic health organisations. Data of people with malaria diagnosed by rapid diagnostic tests during 2017–2018 were extracted from the ICMV registers. Documentation of prescribing a complete course of drugs was used to assess quality. Of 2881 people with malaria, village-based ICMV diagnosed and treated 2279 (79%) people. Overall, 2726 (95%) people received correct drugs in the correct dose and adequate duration appropriate to malaria species, age and pregnancy status while 1285 (45%) people received ‘correct and timely (within 24 h of fever)’ treatment. Children under five years, those with severe malaria, mixed infection and *falciparum* malaria were less likely to receive the correct treatment. When compared to health posts, village-based ICMVs and mobile teams performed better in providing correct treatment and mobile teams in providing ‘correct and timely’ treatment. This calls for ensuring the early presentation of people to health workers within 24 h of undifferentiated fever through health promotion initiatives. Future studies should assess adherence to medication and clinical improvement.

## 1. Introduction

Malaria, a major public health problem, is caused by *Plasmodium* parasites transmitted through infected mosquitoes. It causes acute febrile illness and sometimes resulting in serious life-threatening complications. Half of the world’s population is at risk of malaria infection. Globally in 2017, there were an estimated 219 million people with malaria and 435,000 people died of malaria [1].

World Health Organization has emphasised early diagnosis and prompt treatment within 24–48 h of symptoms to decrease the risk of severe complications and onward transmission of malaria [2]. Early and accurate diagnosis of malaria is possible at primary health care facilities and community settings because of the advent and scaling up of rapid diagnostic test (RDT) kits [1,2].

Southeast Asia region bears the second largest burden of malaria morbidity [3]. Despite declining malaria incidence, Myanmar remains one of the highest malaria burden countries in the Southeast Asia region [4,5,6]. The estimated people with malaria decreased from 2 million in 2010 to 0.1 million in 2017. Estimated deaths because of malaria decreased from 3885 in 2010 to 218 in 2017 [1]. Mortality was high among populations residing along the Thailand and Indian borders, among the internally displaced population in conflict zones and population in ethnic minority regions (henceforth referred to as ethnic communities) [7,8]. Ethnic armed organisations administer most villages in ethnic communities. Government-appointed health staff is usually not present in those villages or village tracts (a group of nearby villages defined by the government for administrative purpose). Permission form ethnic armed organisations are mandatory to deliver health services in ethnic communities besides the government approval.

In ethnic communities (2013), malaria accounted for 18% of all deaths and was the second leading cause of death among under-five children [9,10]. Because of the conflicts and political instability in many ethnic communities, formal health care services by skilled health personnel (medical or paramedical from public or private sector) are not available. Ethnic communities developed ethnic health organisations (EHOs) with trained healthcare workers during the 1990s with the technical assistance of Mae Tao clinic. Mae Tao clinic was established in 1989 in Mae Sot, Thailand, to provide primary healthcare services to underserved populations in conflict-affected areas [11]. These EHOs provide vital services to thousands of vulnerable people through rural health posts and mobile teams as well as trauma care during conflicts. They also train community-based volunteers such as trained birth attendants and community health workers to deliver timely health services. They have deep ties with their communities and remain the sole or preferred provider in these remote, conflict-affected and underserved areas of the country. Geographical access and their role as frontline healthcare providers are critical [9].

Myanmar’s national malaria control program (NMCP) recommends all health service providers including community-based village health volunteers (VHV) to test all undifferentiated fever cases for malaria and to start treatment (if positive) within 24 h of fever onset [12]. A community-based survey in Myanmar in 2015 reported that 44% of people with undifferentiated fever underwent malaria testing within 24 h and a nationwide migrant malaria survey in 2016 revealed that it was only 23% in migrant people [13,14]. In 2015, nationwide malaria data of NMCP indicated that 36% people diagnosed with malaria received treatment from VHV within 24 h of fever onset and 83% received correct treatment from VHV as per national guideline [15,16]. It did not include data from non-governmental organisations and EHOs.

In Myanmar, although the risk for malaria, disease burden and mortality are substantially high among the ethnic communities, there is no published document that assesses the quality of malaria treatment services provided by the EHOs separately. Therefore, we assessed the extent of and factors associated with non-receipt of ‘correct’ and non-receipt of ‘correct and timely’ malaria treatment services provided by EHOs under the Better Health Together (BHT) project in rural hard-to-reach areas of Myanmar.

## 2. Materials and Method

### 2.1. Study Design

This was a cross-sectional study involving secondary data.

### 2.2. Setting

#### 2.2.1. General Setting

Myanmar is located in the Southeast Asia region with 14 states/regions and Nay Pyi Taw union territory. There are 135 ethnicities who reside in the country and many ethnic communities are in the border areas. In some ethnic communities, political tension remains although bilateral ceasefires and the national ceasefire agreement of 2015 have greatly contributed to increased stability and security.

**Figure 1:**
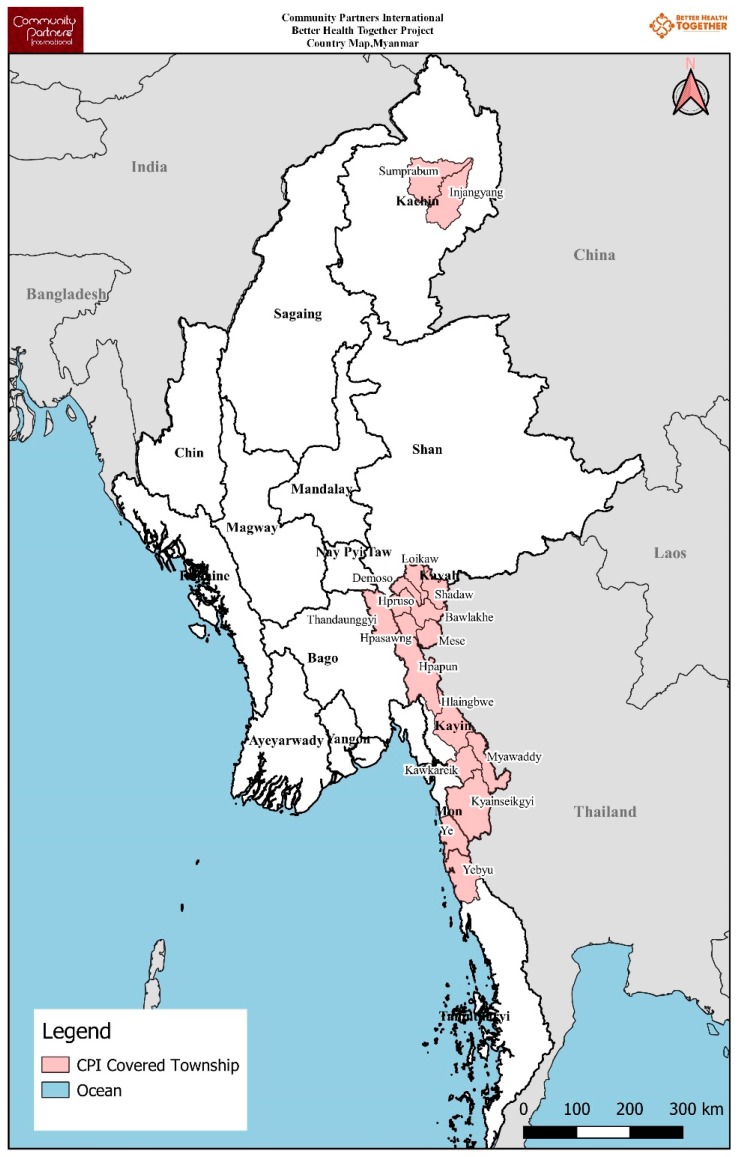
Townships shown in dark red where ethnic health organisations were delivering malaria diagnosis and treatment services under the Better Health Together (BHT) project * in Myanmar (2017–2018). * funded by the Three Millennium Development Goal Fund (3MDG).

Community Partners International (CPI) is a not-for-profit international development organisation dedicated to improving the health and well-being of conflict-affected, underserved and hard-to-reach communities. CPI started the BHT project in 17 townships of Kachin, Kayah, Kayin and Mon State, and Tanintharyi Region in Myanmar in 2017 in collaboration with six EHOs (near the Chinese and Thai border). The project was funded by the Three Millennium Development Goal Fund (3MDG) and implemented in the partnership model in which CPI provides technical support to EHOs to deliver malaria prevention and treatment services with the guidance of NMCP. The project has delivered malaria prevention, diagnosis and treatment services to an estimated 257,700 people through 632 volunteers (see Figure 1 and Table 1).

#### 2.2.2. Malaria Diagnosis and Treatment Provided by EHOs

EHOs deliver health services including malaria testing and treatment through rural health posts, mobile teams and community-based VHV. Integrated community malaria volunteer (ICMV) is a variant of VHV. EHOs trained ICMVs in using ICMV manual (developed by NMCP) containing danger signs, diagnosis and treatment of malaria, signs and symptoms of tuberculosis, health education on dengue, filariasis, STI/HIV and leprosy [4].

To implement the BHT project, EHOs recruited ICMV based on the NMCP’s ICMV criteria: Able to read and write; not too young or too old; recommended by the village health committee; residence in implementing village and willing to volunteer. CPI trained EHO project staff in using ICMV manual. Then EHO project staff conducted multiplier training to volunteers. Each training lasted nine days; seven days for ICMV guideline and two days for recording and reporting formats. EHOs conducted refresher training for volunteers annually.

After training, EHOs assign ICMVs to their respective villages as well as to the health posts and mobile teams to reinforce EHO’s health workers. Village-based ICMVs are responsible to provide services in their resident village and up to three nearby villages depending on the transport condition and their commitment. Their workload varies with the season because of the seasonal trend of malaria and other fever. They receive a fixed honorarium (≈17 USD) per month.

The ICMVs screen all people with undifferentiated fever using RDT for *Plasmodium falciparum *(Pf) and *Plasmodium vivax* (Pv) antigen and treat or refer in accordance with the manual for ICMV. Artemether 20 mg/Lumefantrine tablets are used as schizonticidal drugs in Pf and mixed infection and chloroquine tablets are used in Pv/non-Pf malaria. Primaquine is used as a gametocidal drug for radical curative treatment (see Table 2 and Table 3). Glucose-6-phosphate dehydrogenase (G6PD) deficiency screening is not usually accessible before primaquine administration due to limited resource.

**Figure 2:**
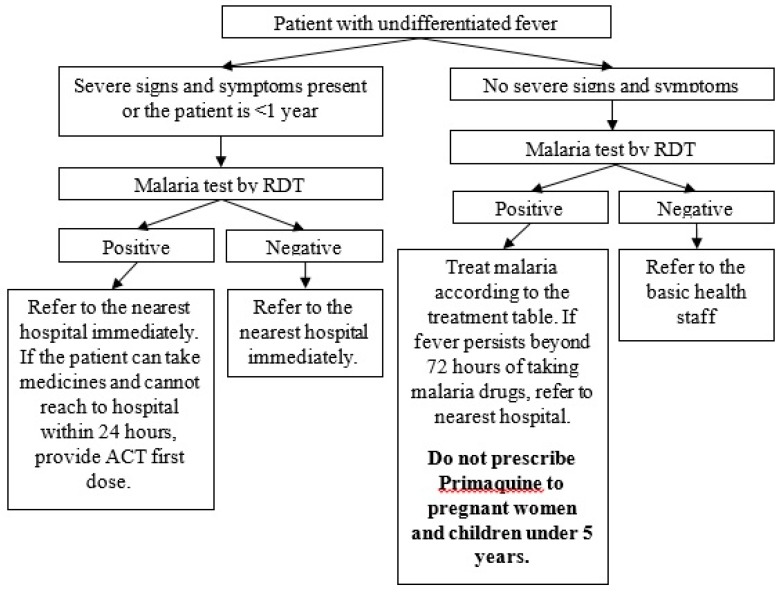
Malaria diagnosis and treatment for village-based health volunteers under ethnic health organisations in the Better Health Together (BHT) project *, Myanmar (2017–2018). RDT: rapid diagnostic test. * funded by the Three Millennium Development Goal Fund (3MDG).

Testing, diagnosis and treatment of malaria are done in one-time contact (see Figure 2, Table 2 and Table 3). ICMVs prescribe a complete course of drugs after confirmation of malaria. Although NMCP recommends follow-up on the third day of treatment in people with Pf malaria, the practice is not followed in all. Patients with severe malaria (see Box 1) are to be referred to the nearest hospital immediately.

Box 1 **Checklist for assessment of severe malaria which is used by village-based health volunteers under Ethnic health organisations in the Better Health Together project, Myanmar (2017–2018).**

If any of the following signs and symptoms is observed, the patient should be referred to the nearest hospital immediately.

- Generalised weakness so that the person is unable to sit, stand or walk without assistance
- Unable to take medicines due to vomiting
- High fever
- Paleness because of severe anaemia
- Jaundice, yellow sclera
- Impaired consciousness
- Confusion
- Agitation
- Drowsiness
- Irritability
- Breathing difficulty, tightness of chest
- Cold and clammy extremities, signs and symptoms of shock
- Reduced or no urine output
- Fits, loss of consciousness
- Passing of dark colour urine, blood tarry stool
- Bleeding from various sites of body

In addition to ICMVs, existing health workers in EHOs also provide malaria testing and treatment in health posts and mobile teams.

#### 2.2.3. Recording, Reporting and Data Verification

ICMVs record testing and treatment data in ICMV register which contains four copies. They keep one copy and send other copies to EHO head offices where data staff single-enter the data using ACCESS software. Then, EHO head offices send one copy to NMCP and one copy to CPI.

Monitoring and evaluation (M&E) supervisors of EHOs check data completeness, accuracy, reliability and timeliness at the time of collecting malaria carbonless registers from ICMVs. They also perform random checking for data consistency between the electronic data set and malaria carbonless registers monthly. CPI M&E team performs routine data quality assessment at EHO offices and volunteer residence quarterly. CPI M&E team members perform data verification by recounting the reported numbers in the source documents and random checking of indicators across electronic database and source documents. In addition, they also assess volunteers’ knowledge of indicator definition, reporting guidelines, forms and formats.

### 2.3. Study Population

We included all people with confirmed malaria (tested positive using RDT—all age groups and pregnant women) that were recorded in the ICMV register maintained at health posts, mobile teams and village-based ICMVs managed by six EHOs in ethnic communities under BHT project in Myanmar between 2017 and 2018.

### 2.4. Data Variables and Source of Data

Data in electronic format were extracted from the ACCESS database (see Annex S1). Data variables used in this study were EHO identifier, state/region, service delivery type (health posts, mobile team and village-based ICMV), age group, gender, presentation within 24 h of fever onset (yes/no), RDT result (*Plasmodium falciparum*—Pf, *Plasmodium vivax*—Pv, mixed), pregnancy (if female), severe malaria (yes/no), referred (yes/no), and number of artesunate combination tablets, chloroquine and primaquine tablets dispensed.

### 2.5. Operational Definitions

‘Correct’ malaria treatment was defined as the documentation of dispensing the correct schizonticidal and gametocidal drugs in the correct dose and adequate duration appropriate to species, age and pregnancy status (see Table 2). For infants and people with severe malaria, if the patient was referred (with or without the first dose), we considered the treatment to be ‘correct’. If complete treatment was provided (in addition to the first dose) and the patient was not referred, we considered that treatment was ‘not correct’. Prescription of a complete course of drugs (number of each type of tablet) at the time of first dose prescription was assumed as a correct treatment since there was no follow-up data. In deciding whether treatment was correct, the timing of treatment (within 24 h of fever or not) was not considered.

‘Correct and timely’ treatment was defined as the patient receiving the correct treatment and presenting within 24 h of fever onset. Service providers decided whether the patients presented within 24 h of fever onset or not by asking them when the fever started.

### 2.6. Data Analysis

The electronic data set was imported into STATA (version 12.1 STATA Corp., College Station, TX, USA) for analysis. Frequencies and proportions (95% confidence interval (CI)) were used to assess the key outcomes. Association of baseline characteristics with non-receipt of correct treatment and non-receipt of ‘correct and timely’ treatment were assessed using unadjusted and adjusted prevalence ratios and 95% CIs. Modified Poisson regression with robust variance estimation was used for the adjusted analysis and all the available variables were included. Because of the large sample size, factors were assessed for significance by first looking at the programmatically significant association (adjusted prevalence ratio >1.5 or <0.7) and then looking at the statistical significance (*p <* 0.05).

### 2.7. Ethics

Ethics approval was sought from Institutional Technical and Ethical Review Board, University of Public Health, Yangon, Myanmar (UPH-IRB 2019/Research/39) and Ethics Advisory Group of the International Union against Tuberculosis and Lung Disease (The Union), Paris, France (EAG no. 02/19). Administrative approval was obtained from the NMCP. As this study involved routinely collected secondary data, the waiver for informed consent was sought and approved by the ethics committees. Name of the EHO was not used while reporting the data to maintain confidentiality.

## 3. Results

### 3.1. Baseline Characteristics

A total of 169,904 people with undifferentiated fever were tested for malaria and 3018 (1.8%) were positive. Of 3018 with malaria, we excluded 137 that had incomplete data. We have summarised the baseline characteristics of the 2881 people included in the study in Table 4. Among 2881, 1045 (36.3%) were female and 24 (2.3% of female) were pregnant. Of the 2881 people, 1024 (35.6%) had Pf, 1730 (60%) had Pv and 127 (4.4%) had mixed infection. Village-based ICMVs diagnosed and treated 2279 (79.1%) people with malaria.

### 3.2. Quality of Malaria Treatment and Associated Factors

**Figure 3:**
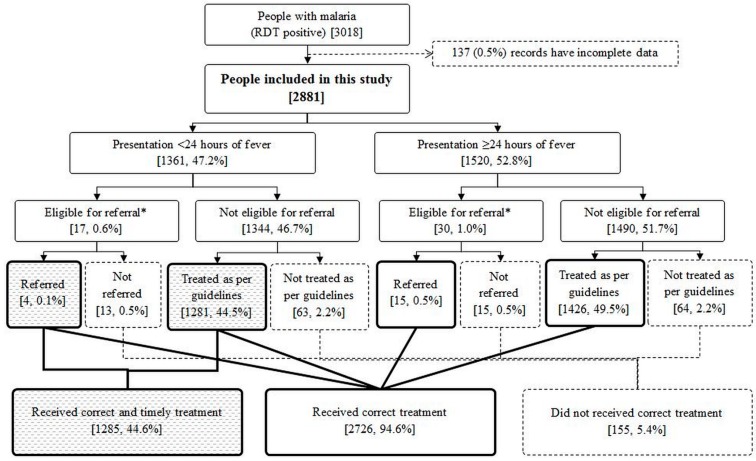
Management of people with confirmed malaria by EHO in rural hard-to-reach areas under BHT project in Myanmar, 2017–2018. EHO: Ethnic Health Organisation; BHT: Better Health Together funded by the Three Millennium Development Goal Fund (3MDG); RDT: rapid diagnostic test; * Infants or people with severe malaria.

Of the 2881 people, 1361 (47.2%, 95% CI: 45.4–49.1) presented within 24 h of fever onset. A total of 2726 (94.6%, 95% CI: 93.7, 95.3) people received correct treatment while 1285 (44.6%, 95% CI: 42.8, 46.4) people received ‘correct and timely’ treatment (Figure 3). Among pregnant women (n = 24), 23 (95.8%) received correct treatment and six (25.0%) received ‘correct and timely’ treatment (data not shown).

The documented reasons for not providing correct treatment (n = 155) were: (i) Not referring infant and severe malaria cases according to the guideline (28, 18.1%); (ii) no or incorrect prescription of schizonticidal drugs (47, 30.3%); (iii) no or incorrect prescription of gametocidal drug (51, 32.9%); and (iv) no or incorrect prescription of both drugs (29, 18.7%). Among no or incorrect prescription (n = 127), 18 (14.2%) were due to stock out (data not shown). “No prescription” means not prescribing the schizonticidal or gametocidal drugs because of stock out or reluctance to provide primaquine while ‘incorrect prescription’ means prescribing incorrect drugs, doses or duration.

We have depicted the factors associated with not receiving the correct treatment in Table 5. In adjusted analysis, compared to over 15 years, infants were 21 times and children in 1–4-year and 10–14-year age groups were two times less likely to receive the correct treatment. People with severe malaria were five times less likely to receive correct treatment compared to people without severe malaria. People with Pf were two times and with mixed infection were five times less likely to receive correct treatment compared to those with Pv infection. Among service delivery types, mobile team and village-based ICMVs were significantly more likely to provide correct treatment than health posts.

We have depicted the factors associated with not receiving ‘correct and timely’ treatment in Table 6. Compared to health posts, mobile teams were significantly more likely to provide ‘correct and timely’ treatment.

## 4. Discussion

In this first study on quality of malaria treatment in ethnic communities of Myanmar, most people with malaria received correct drugs in the correct dose and adequate duration appropriate to species, age and pregnancy status. However, the timeliness of correct treatment was seen in half, and this was due to presentation after 24 h of fever onset. Infants, those with severe malaria, Pf infection and mixed infection were less likely to receive the correct treatment. Village-based ICMVs were more likely to provide the correct treatment. Mobile teams were more likely to provide correct as well as ‘correct and timely’ treatment.

### 4.1. Strengths and Limitations

A study based on the nationwide malaria data of NMCP (2015) had assessed the correctness of malaria treatment. The number of tablets dispensed according to the type of malaria, age group and pregnancy status was used to assess the correctness of treatment [15]. The definition used in our study was more rigorous and we also used ‘eligibility for referral’, ‘referred or not’ and timeliness (among those with correct treatment) to assess the quality of malaria. We were able to analyse the reasons for incorrect treatment as EHOs had good documentation practices.

There were some limitations. The risk factors assessed were limited to the variables available in records and other socio-demographic, programmatic and health system level factors were not assessed. Second, the study assessed only the prescription of the complete course of drugs (number of each type of tablet) at the time of first dose prescription. It did not include follow-up data on: (1) If people sought care elsewhere; (2) if people completed their course; (3) clinical outcomes. Finally, referred people could not be assessed for receipt of complete treatment.

### 4.2. Key Findings and Implications

Limitations notwithstanding, the study has some key findings. Provision of correct treatment was satisfactory. The correctness of treatment given by VHVs was 83% nationally in 2015 (did not include data from ethnic communities) while it was 95% in our study [15,16]. We speculate continuous supervision of village-based ICMV, refresher training and hands-on training to new recruits by EHOs as the reasons [17].

The correctness can be further improved by appropriate management of severe malaria and malaria in children (especially infants). This was mostly due to lack of referral among this sub-group despite being eligible and has to be addressed in refresher training. EHOs need to strengthen the referral system in hard to reach villages. Reason for non-receipt of correct treatment among people with mixed infections is unknown. Although drug stock-out was minimal, proper supply chain management can further improve the correctness of treatment. According to EHOs’ reports, drug stock-out was common in hard to reach villages because of unpredictable transportation time in the rainy season.

Despite most receiving the correct treatment, it was not timely in half because of presenting after 24 h of fever onset. This finding is similar to the previous study based on the nationwide malaria data of NMCP [15]. The reasons from the patient side for delayed presentation have been studied elsewhere among migrant populations [13,18]. These are inappropriate health care seeking, self-medication, not giving importance to fever, transportation difficulty (if allotted volunteer lived in another village), uninformed about the volunteer or his/her activities and lack of symptomatic treatment from a volunteer [13]. From the volunteer side, lack of practices for malaria testing in all undifferentiated fever cases, and apprehension regarding paper workload and wastage of test kits have been reported [13]. To improve the knowledge of community on the importance of early diagnosis and treatment and danger of self-medication, information, education and communication materials and strategy should be reviewed and revised according to local context such as using ethnic language. ICMV should improve the visibility of available services by posters or awareness sessions. Encouraging ICMV to test all undifferentiated fever is important for early diagnosis.

Mobile teams and health posts’ contribution to diagnosed patients was low. Mobile teams were significantly associated with providing a better quality of treatment when compared to health posts. We are unsure of the reason for the poor performance of health posts (was poorer than village-based ICMVs) in providing correct treatment (irrespective of timeliness). While addressing these, one must also review the role of ICMVs as they largely contribute to the diagnosis of malaria [19]. Though they provide correct treatment, their role in providing ‘correct and timely’ treatment has scope to improve.

No or incorrect prescription of gametocidal drug contributed to a significant portion of non-receipt of correct treatment. According to narrative reports from EHOs, ICMVs had concerns about prescribing primaquine to people with unknown G6PD status. G6PD deficiency is common in Myanmar, 19.8% of people with malaria have G6PD deficiency [20]. NMCP recommends G6PD deficiency screening before primaquine administration. Primaquine is contraindicated in severe G6PD deficiency and weekly primaquine was recommended (during study period) for mild to moderate deficiency [5,12]. However, implementing this is impractical in ethnic communities as most have unknown G6PD status. Since Pv and mixed infections contribute to more than two-thirds of malaria in this setting, scaling up of G6PD testing is important. We recommend that once G6PD testing is scaled up, guidelines to treat Pv and mixed malaria infection in those with unknown G6PD status should be specified as ‘receipt of appropriate schizonticidal drug followed by referral to nearest health facility like rural health centre or station hospital for G6PD testing’.

Follow-up information after prescribing drugs or referral was not collected. Failure to comply with a full course of treatment could impact patient outcome. Therefore, this information is useful for programme design improvement, and routine data collection should contain follow-up information to assess compliance with treatment. Given the concerns regarding artemisinin resistance, future studies should assess adherence to medication and clinical improvement.

Most people with malaria were adults, despite malaria being more prevalent among children. Possible reasons such as missing malaria cases among children or an epidemiological shift in these communities because of successful preventive interventions for malaria have to be explored.

To conclude, the correctness of malaria treatment provided by EHOs under the BHT project was satisfactory. This can be moved closer to 100% in ethnic communities by improving the management of under-five children, severe malaria and mixed infection. However, timeliness of treatment needs to be improved. This calls for ensuring the early presentation of people to health workers within 24 h of undifferentiated fever through health promotion initiatives. Role of ICMVs has to be maximised for this purpose as a large proportion of people with malaria are being detected by village-based ICMVs. Addressing these issues among ethnic communities are key to eliminate malaria in Myanmar by 2030 [12].

## Figures and Tables

**Table 1:** Townships where ethnic health organisations are delivering malaria diagnosis and treatment services under the Better Health Together (BHT) project in Myanmar (2017–2018).

S. No.	State/Region	Project Implementation Townships	Other Malaria-Related Organisations ^	Total ICMVs	ICMVs Trained by BHT Project	Estimated Population Covered by BHT Project
1.	Kachin	Injangyang	HPA	51	6	1500
2.	Kachin	Sumprabum	HPA/MAM	39	39	7030
3.	Kayah	Bawlake	NMCP	37	13	3310
4.	Kayah	Demoso	NMCP/MAM	146	49	29,210
5.	Kayah	Hpasawng	NMCP/MAM	85	22	4140
6.	Kayah	Hpruso	NMCP/MAM	173	60	13,950
7.	Kayah	Loikaw	NMCP	116	64	30,780
8.	Kayah	Mese	NMCP	26	14	5230
9.	Kayah	Shadaw	NMCP	49	16	4320
10.	Kayin	Hlaingbwe	NMCP/ARC/KDHW/MHAA/SCI/SMRU	709	52	13,040
11.	Kayin	Hpa-An		35	35	10,500
12.	Kayin	Kawkareik	NMCP/ARC/KDHW/MAM/SMRU	409	36	14,410
13.	Kayin	Kyainseikgyi	NMCP/ARC/KDHW/MAM	658	160	55,530
14.	Kayin	Myawaddy	NMCP/ARC/SMRU	239	20	42,170
15.	Kayin	Thandaunggyi	NMCP/MAM	355	16	10,430
16.	Mon	Ye	NMCP/ARC/IOM/KDHW/MAM	180	22	10,710
17.	Tanintharyi	Yebyu	NMCP/ARC/KDHW/MAM/PSI/URC	245	8	1440
	**Total**			**3552**	632 *	**257,700**

ICMV—integrated community malaria volunteers; EHO: Ethnic Health Organisation; BHT: Better Health Together funded by the Three Millennium Development Goal Fund (3 MDG); CPI: Community Partners International; * among 632 volunteers, EHO A, EHO B, EHO C, EHO D, EHO E and EHO F have 71, 220, 45, 124, 117 and 55 volunteers, respectively. ^ Other malaria-related organisations (HPA = Health Poverty Action, MAM= Medical Action Myanmar, NMCP = National Malaria Control Programme, ARC = American Refugee Committee, KDHW = Kayin Department of Health and Warfare, MHAA = Myanmar Health Assistant Association, SCI = Save the Children International, SMRU = Shoklo Malaria Research Unit, IOM = International Organization for Migration, PSI = Population Service International, URC = University Research Co., LLC).

**Table 2:** Plasmodium falciparum malaria and mixed infection* treatment guidelines for village-based health volunteers under Ethnic health organisations in the Better Health Together project, Myanmar (2017–2018).

Age Group (yr)	Artemether 20 mg/Lumefantrine 120 mg Tablet						Primaquine 7.5 mg Tablet
	1st Day		2nd Day		3rd Day		
	1st Dose	2nd Dose	3rd Dose	4th Dose	5th Dose	6th Dose	Stat Dose
<1	1/2	1/2	1/2	1/2	1/2	1/2	-
1–4	1	1	1	1	1	1	-
5–9	2	2	2	2	2	2	2
10–14	3	3	3	3	3	3	4
≥15	4	4	4	4	4	4	6

Mixed infection*—similar to above but for ‘stat dose’ of Primaquine on the first day, followed by weekly once for eight weeks.

**Table 3:** Plasmodium vivax/non-plasmodium falciparum malaria* treatment guidelines for village-based health volunteers under Ethnic health organisations in the Better Health Together project, Myanmar (2017–2018).

Age Group (yr)	Chloroquine 150 mg Tablet			Primaquine 7.5 mg Tablet
	1st Day	2nd Day	3rd Day	Stat Dose on 4th Day, Weekly Once for 8 Weeks
<1	1/3	1/3	1/3	-
1–4	1.5	1.5	1	-
5–9	2	2	1	2
10–14	3	3	1.5	4
≥15	4	4	2	6

* Do not prescribe Primaquine to pregnant women and children under 5 years. If fever persists beyond 72 h of taking malaria drugs, refer to the nearest hospital. Be aware of the milligram composition of Primaquine since 15 mg tablets are also available.

**Table 4:** Baseline characteristics of people with confirmed malaria diagnosed by EHO in rural hard-to-reach areas under BHT project in Myanmar, 2017–2018.

Variables		n	(%)
Total		2881	(100.0)
**Age in years**			
	<1	25	(0.9)
	1–4	338	(11.7)
	5–9	442	(15.3)
	10–14	387	(13.4)
	≥15	1689	(58.6)
**Gender**			
	Male	1836	(63.7)
	Female	1045	(36.3)
**RDT result**			
	Pf	1024	(35.6)
	Pv	1730	(60.0)
	Mixed	127	(4.4)
**Severe malaria**			
	Yes	22	(0.8)
	No	2859	(99.2)
**EHO**			
	EHO A	912	(31.7)
	EHO B	121	(4.2)
	EHO C	202	(7.0)
	EHO D	491	(17.0)
	EHO E	886	(30.8)
	EHO F	269	(9.3)
**State/Region**			
	Kachin	202	(7.0)
	Kayah	129	(4.5)
	Kayin	2279	(79.1)
	Mon	65	(2.3)
	Tanintharyi	206	(7.2)
**Service delivery type**			
	Health posts^	202	(7.0)
	Mobile	129	(4.5)
	Village-based ICMV	2279	(79.1)
	Missing	536	(9.4)

EHO: Ethnic Health Organisation; BHT: Better Health Together funded by the Three Millennium Development Goal Fund (3MDG); RDT: rapid diagnostic test; Pf: *Plasmodium falciparum*; Pv: *Plasmodium vivax*; ICMV: integrated community malaria volunteers. ^ These are manned by EHO trained basic health staff.

**Table 5:** Factors associated with not receiving correct treatment as per national guidelines among people with confirmed malaria diagnosed by EHO in rural hard-to-reach areas under BHT project in Myanmar, 2017–2018.

Variables	N	Not Correct Treatment		PR	(95%CI)	aPR	(95%CI)
		n	(%)				
Total	2881	155	(5.4)	-		-	
**Age in years**							
<1	25	19	(76.0)	19.75	(14.27–27.32)	21.17	(12.45–35.99) *
1–4	338	25	(7.4)	1.92	(1.23–3.00)	2.49	(1.59–3.89) *
5–9	442	21	(4.8)	1.23	(0.76–2.00)	1.53	(0.96–2.43)
10–14	387	25	(6.5)	1.68	(1.07–2.63)	1.71	(1.13–2.60) *
≥15	1689	65	(3.9)	Ref			
**Gender**							
Male	1836	99	(5.4)	1.01	(0.73–1.38)	1.17	(0.85–1.60)
Female	1045	56	(5.4)	Ref			
**Severe malaria**							
Yes	22	9	(40.9)	8.01	(4.73–13.56)	5.21	(2.49–10.93) *
No	2859	146	(5.1)	Ref			
**RDT result**							
Pf	1024	62	(6.1)	1.90	(1.34–2.72)	1.84	(1.29–2.63) *
Pv	1730	55	(3.2)	Ref			
Mixed	127	38	(29.9)	9.41	(6.49–13.65)	5.49	(3.57–8.44) *
**EHO**							
EHO A	912	50	(5.5)	4.05	(2.17–7.55)	2.57	(1.37–4.82) *
EHO B	121	12	(9.9)	7.32	(3.37–15.93)	2.35	(0.84–6.55)
EHO C	202	15	(7.4)	5.48	(2.61–11.53)	4.82	(2.35–9.90) *
EHO D	491	63	(12.8)	9.47	(5.16–17.39)	7.11	(3.82–13.24) *
EHO E	886	12	(1.4)	Ref			
EHO F	269	3	(1.1)	0.82	(0.23–2.9)	0.00	(0.00–0.00)
**State/Region**							
Kachin	202	15	(7.4)	1.38	(0.82–2.31)	- **	
Kayah	129	14	(10.9)	2.01	(1.19–3.39)		
Kayin	2279	123	(5.4)	Ref			
Mon	65	0	(0.0)	-			
Tanintharyi	206	3	(1.5)	0.27	(0.09–0.84)		
**Service delivery type**							
Health posts ^	923	49	(5.3)	Ref			
Mobile	139	1	(0.7)	0.14	(0.02–0.97)	0.10	(0.01–0.73) *
Village-based ICMV	1283	73	(5.7)	1.07	(0.75–1.52)	0.50	(0.30–0.83) *
Missing	536	32	(6.0)	1.12	(0.73–1.73)	0.61	(0.36–1.04)

EHO: Ethnic Health Organisations; BHT: Better Health Together funded by the Three Millennium Development Goal Fund (3MDG); RDT: rapid diagnostic test; Pf: *Plasmodium falciparum*; Pv: *Plasmodium vivax*; ICMV: integrated community malaria volunteers; PR: prevalence ratio; aPR: adjusted prevalence ratio; CI: confidence interval; * aPR programmatically significant (>1.5 or <0.7) and statistically significant (*p *< 0.05); ** excluded by the model due to collinearity; ^ these are manned by EHO trained basic health staff.

**Table 6:** Factors associated with not receiving ‘correct and timely’ treatment as per national guidelines among people with confirmed malaria diagnosed by EHO in rural hard-to-reach areas under BHT project in Myanmar, 2017–2018.

Variables	N	Not ‘Correct and Timely’ Treatment		PR	(95%CI)	aPR	(95%CI)
		n	(%)				
Total	2881	1596	(55.4)	-		-	
**Age in years**							
<1	25	21	(84.0)	1.50	(1.26–1.79)	1.47	(1.2–1.79)
1–4	338	193	(57.1)	1.02	(0.92–1.13)	1.09	(0.99–1.2)
5–9	442	229	(51.8)	0.93	(0.84–1.02)	0.94	(0.86–1.04)
10–14	387	209	(54.0)	0.97	(0.87–1.07)	0.96	(0.87–1.06)
≥15	1689	944	(55.9)	Ref			
**Gender**							
Male	1836	1044	(56.9)	1.08	(1.00–1.15)	1.08	(1.01–1.15)
Female	1045	552	(52.8)	Ref			
**Severity**							
Severe	22	22	(100.0)	1.90	(1.84–1.97)	1.66	(1.42–1.93)*
Not severe	2859	1574	(55.1)	Ref			
**RDT result**							
Pf	1024	621	(60.6)	1.19	(1.11–1.27)	1.22	(1.14–1.31)
Pv	1730	881	(50.9)	Ref			
Mixed	127	94	(74.0)	1.45	(1.30–1.63)	1.35	(1.21–1.52)
**EHO ID**							
EHO A	912	558	(61.2)	1.28	(1.18–1.40)	1.14	(1.03–1.27)
EHO B	121	90	(74.4)	1.56	(1.38–1.77)	1.46	(0.95–2.26)
EHO C	202	59	(29.2)	0.61	(0.49–0.77)	0.59	(0.47–0.74)
EHO D	491	349	(71.1)	1.49	(1.37–1.63)	1.45	(1.32–1.6)
EHO E	886	422	(47.6)	Ref			
EHO F	269	118	(43.9)	0.92	(0.79–1.07)	1.06	(0.68–1.66)
**State/Region**							
Kachin	202	59	(29.2)	0.50	(0.40–0.62)	1.00	
Kayah	129	96	(74.4)	1.28	(1.15–1.43)	1.10	(0.73–1.67)
Kayin	2279	1324	(58.1)	Ref			
Mon	65	23	(35.4)	0.61	(0.44–0.85)	0.74	(0.47–1.18)
Tanintharyi	206	94	(45.6)	0.79	(0.67–0.92)	0.94	(0.58–1.51)
**Service delivery type**							
Health post ^	923	569	(61.7)	Ref			
Mobile	139	61	(43.9)	0.71	(0.59–0.86)	0.68	(0.56–0.82) *
Village-based ICMV	1283	661	(51.5)	0.84	(0.78–0.90)	0.79	(0.71–0.88)
Missing	536	305	(56.9)	0.92	(0.84–1.01)	0.91	(0.82–1.01)

EHO: Ethnic Health Organisations; BHT: Better Health Together funded by the Three Millennium Development Goal Fund (3MDG); RDT: rapid diagnostic test; Pf: *Plasmodium falciparum*; Pv: *Plasmodium vivax*; ICMV: integrated community malaria volunteers; PR: prevalence ratio; aPR: adjusted prevalence ratio; CI: confidence interval; * aPR programmatically significant (>1.5 or <0.7) and statistically significant (*p* < 0.05); ** excluded by the model due to collinearity; ^ these are manned by EHO trained basic health staff.

## Supplementary Materials

The following are available online at https://www.mdpi.com/2414-6366/4/4/140/s1, Annex S1: Dataset used for analysis including codebook.
